# Neutralization of Omicron XBB.1 by booster vaccination with BA.4/5 monovalent mRNA vaccine

**DOI:** 10.1038/s41421-023-00609-0

**Published:** 2024-01-09

**Authors:** Hui Zhao, Na-Na Zhang, Suad Hannawi, Ai-Ru Zhu, Xiao-Chuan Xiong, Yi-Jiao Huang, Dan-Dan Yu, Can-Jie Chen, Jun Dai, Alaa Abuquta, Bo Ying, Jin-Cun Zhao, Cheng-Feng Qin

**Affiliations:** 1 State Key Laboratory of Pathogen and Biosecurity, Beijing Institute of Microbiology and Epidemiology, Academy of Military Medical Sciences, Beijing, China; 2 School of Medicine, Tsinghua University, Beijing, China; 3 Internal Medicine Department, Al Kuwait-Dubai Hospital, Emirates Health Services (EHS), Ministry of Health and Prevention, Dubai, UAE; 4 State Key Laboratory of Respiratory Disease, National Clinical Research Center for Respiratory Disease, Guangzhou Institute of Respiratory Health, the First Affiliated Hospital of Guangzhou Medical University, Guangzhou, Guangdong China; 5 Suzhou Abogen Biosciences, Suzhou, Jiangsu China; 6 Health and Quarantine Laboratory, Guangzhou Customs District Technology Centre, Guangzhou, Guangdong China; 7 Guangzhou National Laboratory, Guangzhou International Bio Island, Guangzhou, Guangdong China; 8 Research Unit of Discovery and Tracing of Natural Focus Diseases, Chinese Academy of Medical Sciences, Beijing, China

**Keywords:** Immunology, Biological techniques

Dear Editor,

COVID-19 remains a global health threat due to the sequential emergence of novel variants with immune escape capabilities. The predominant circulating variant worldwide has evolved from the prototype, Alpha, Beta, Delta, into Omicron and its subvariants^1,2^, especially the newly emerged XBB descendant lineages. The most updated version of licensed bivalent vaccines contained mRNAs encoding the Spike (S) protein of both prototype and BA.4/5, while the inclusion of ancestral strain antigen was evidenced to induce immune imprinting and lead to reduced vaccine efficacy against the circulating XBB lineage^3–5^. Recently, WHO and FDA have recommended to remove the prototype strain antigen in future formulations of COVID-19 vaccine. However, the immunogenicity and protection of an updated monovalent mRNA vaccine in pre-immune populations remains unknown.

**Fig. 1:**
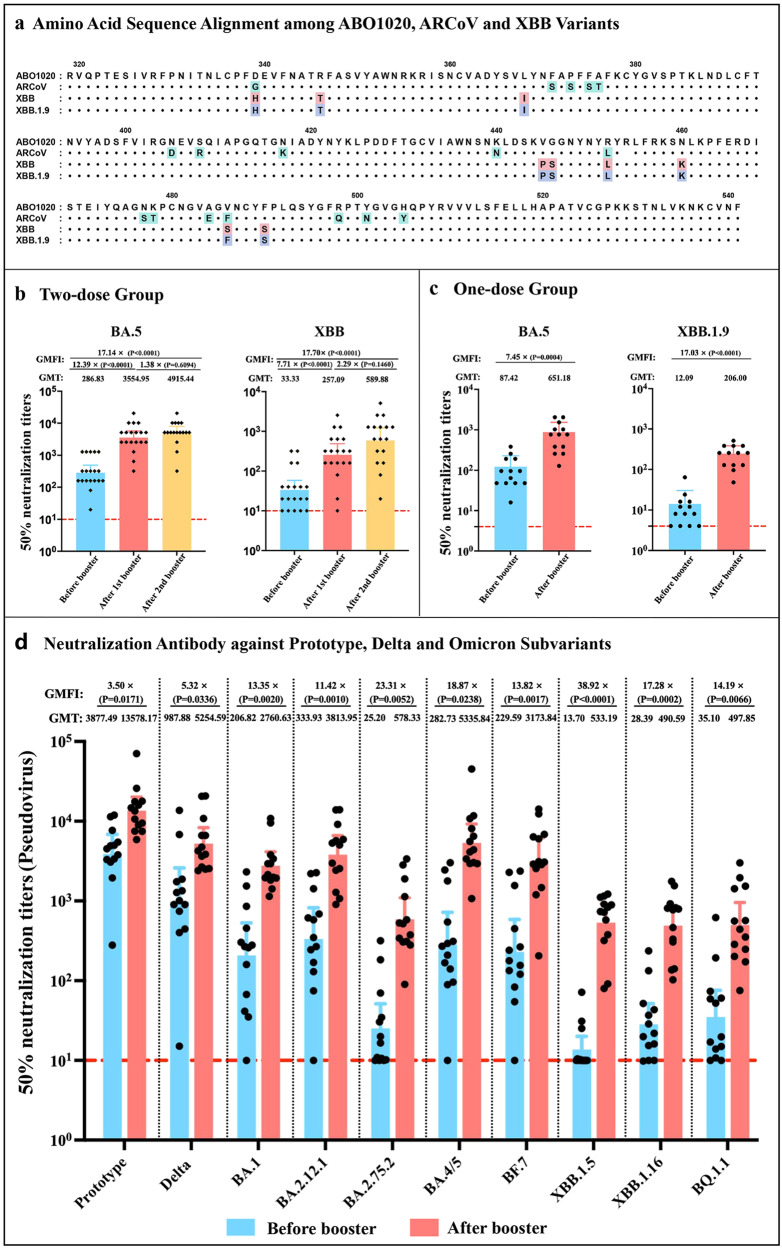
Neutralizing responses with the monovalent BA.4/5 mRNA vaccine booster. **a** Mutations in amino acids of the RBD of S protein edcoded by the monovalent BA.4/5 mRNA vaccine (ABO1020), ARCoV, XBB and XBB.1.9. The amino acid differences in variant sequences from those in BA.4/5 are highlighted. **b** 50% authentic virus neutralization titers against Omicron subvariants BA.5 and XBB after two-dose booster of ABO1020. The neutralizing antibody titers were assessed on day 0 before booster and day 28 after each booster (1st and 2nd boost), respectively. **c** 50% authentic virus neutralization titers against Omicron subvariants BA.5 and XBB.1.9 after a single booster of ABO1020. The neutralizing antibody titers were assessed on day 0 before booster and day 14 after booster, respectively. **d** 50% pseudovirus neutralization titers against prototype strain, Delta and Omicron subvariants BA.1, BA.2.12.1, BA.2.75.2, BA.4/5, BF.7, XBB.1.5, XBB.1.16 and BQ.1.1 after a single booster of ABO1020. Each dot represents a participant. Average antibody levels are represented by GMT values. The GMFI values in neutralizing titers before and after booster are shown. Statistical significance was determined using the unpaired *t*-test (**b**, **c**) or multiple unpaired *t*-tests (**d**) between before and after booster. The dashed horizontal line represents the lower limit of detection.

Previously, we have developed a monovalent mRNA vaccine (termed ARCoV) targeting the receptor binding domain (RBD) of prototype SARS-CoV-2^6^. ABO1020 is an updated version of ARCoV, which encodes the RBD of Omicron BA.4/5. Compared with the circulating XBB.1 lineages and ARCoV, ABO1020 contained 9 and 17 amino acid substitutions, respectively (Fig. 1a). This BA.4/5 monovalent mRNA vaccine ABO1020 is now in the final stage of international, multicenter, phase 3 trials (ClinicalTrials.gov Identifier: NCT05636319).

Herein, to determine the neutralizing antibodies against BA.4/5 and XBB.1 variants, sera from a cohort of volunteers who have received two doses (28-day interval booster) of ABO1020 booster (15 ug) were collected. All these participants are healthy adults aged 18 years and older who have previously completed a full course of 2 or 3 doses of COVID-19 inactivated vaccine (Supplementary Table S1). Similar to previous findings^7^, ABO1020 was safe and well tolerated, and no serious adverse events occurred after the 1st or 2nd booster. The neutralizing antibodies against BA.5 and XBB were determined by authentic virus assay as previously described^8^. The protocol and informed consent were approved by Ministry of Health and Prevention Research Ethics Committee of AL Kuwait Hospital (MOHAP/DXB-REC/SOO/No.80/2022). This study was conducted in accordance with the principles of the Declaration of Helsinki and Good Clinical Practice.

As expected, all subjects (*n* = 19) produced high levels of BA.5 neutralizing antibodies on day 28 after the 1st booster with ABO1020, and the geometric mean titer (GMT) of neutralizing antibodies reached 3554, with the neutralizing geometric mean fold increase (GMFI) of 12.3 than before booster. On day 28 after the second booster, the neutralizing antibodies against BA.5 further increased to 4915 (GMFI 17.1). Most importantly, the neutralizing antibodies titers against XBB reached 257 (GMFI 7.7) and 589 (GMFI 17.7) after 1st and 2nd booster, respectively (Fig. 1b). Remarkably, the seropositivity rate against XBB increased from 73.7% to 94.7% after the first booster, respectively (Supplementary Fig. S1a).

As the 2nd booster with ABO1020 failed to significantly increase the neutralizing antibody titers against either BA.5 or XBB (Fig. 1b; Supplementary Fig. S2a), we further assayed the impact of a single booster with ABO1020 with another cohort of volunteers (*n* = 13) (Supplementary Table S2). Similar to above results, a single booster with ABO1020 induced high level of neutralizing antibodies against BA.5 (GMT 651, GMFI 7.4) as well as XBB.1.9 (GMT 206, GMFI 17.0) 14 days post booster (Fig. 1c; Supplementary Fig. S2b). Meanwhile, the seropositivity rate of XBB.1.9 increased from 69.2 to 100% (Supplementary Fig. S1b).

Finally, the neutralizing antibody titers against prototype, Delta and Omicron subvariants (BA.1, BA.2.12.1, BA.2.75.2, BA.4/5, BF.7, XBB.1.5, XBB.1.16, BQ.1.1) were detected by pseudovirus neutralization assay in one dose group (Supplementary Table S3). As shown in Fig. 1d and Supplementary Fig. S3, a single booster with ABO1020 could readily induce production of neutralizing antibodies against all tested variants. Especially, the neutralizing antibody titers against XBB.1.5 increased by the highest fold (GMFI 38.9), followed by those against BA.2.75.2 and BA.4/5 (GMFI 23.3 and 18.8, respectively), while the antibody titers against the original strain only increased by 3.50 times. Together, all these results demonstrate that a single- or two-dose booster of ABO1020 induced broad and potent neutralizing antibodies against multiple variants including the XBB.1 variants, with reduced induction of immune imprinting.

The immunogenicity of bivalent mRNA vaccines has been well documented in different populations, while they failed to neutralize XBB subvariants, including XBB.1 and XBB.1.1, with peak titers against the prototype strain^4,9,10^. Our present study confirmed that a single booster with a BA.4/5 monovalent mRNA vaccine in different populations was sufficient to induce protective antibodies against the circulating strains including XBB.1 lineage. Although the population size is limited, all participants at different immune or infection statues showed 100% seroconversion, and the high neutralizing antibodies from authentic virus assay would probably confer significant protection against the circulating XBB.1 variant. As a monovalent vaccine targeting XBB is not available in a short time, the use of a monovalent BA.4/5 mRNA vaccine provides an accessible strategy in response to the ongoing COVID-19 pandemic. Whether other monovalent BA.4/5 mRNA vaccines targeting the full S protein have similar effects deserves immediate investigation.

### Supplementary information

Supplementary Information
