# Supplementary material for: Neutralization of Omicron XBB.1 by booster vaccination with BA.4/5 monovalent mRNA vaccine
Source: Cell Discov. 2024 Jan 9;10:7. doi: 10.1038/s41421-023-00609-0 (PMC10774259; doi:10.1038/s41421-023-00609-0)
Supplement: Supplementary file 1 — Supplementary Information [file 41421_2023_609_MOESM1_ESM.pdf]

Supplementary Appendix

Supplementary Materials Table of Contents

Materials and Methods 2

Acknowledgements 4

Supplementary Figures and Tables 5

Supplementary Fig. S1 5

Supplementary Fig. S2 6

Supplementary Fig. S3 7

Supplementary Table S1 8

Supplementary Table S2 9

Supplementary Table S3 10

Supplemental references 11

Materials and Methods

Serum samples:

In this randomized, double-blind, placebo-controlled clinical study of SARS-CoV-2 variant (BA.4/5) mRNA vaccine (ABO1020) in healthy subjects aged 18 years and older who previously received two or three doses of COVID-19 inactivated vaccine (ClinicalTrials.gov Identifier: NCT05636319), two immunization strategies were used, two-dose (28-day interval booster) and single booster. In two-dose booster subjects, serum samples were collected just prior to and 28 days post each booster with ABO1020. In single booster subjects, serum samples were collected just prior to and 14 days after booster with ABO1020. Written informed consent was obtained from each participant before enrolment. The protocol and informed consent were approved by Ministry of Health and Prevention Research Ethics Committee of AL Kuwait Hospital (MOHAP/DXB-REC/SOO/No.80/2022). This study was conducted in accordance with the principles of the Declaration of Helsinki and Good Clinical Practice.

Plaque reduction neutralization test:

Neutralizing antibodies titers of serum samples were measured by plaque reduction neutralization test with the BA.5 and XBB variants. Briefly, cell suspension solution containing $0.6\text{-}1\times10^6$ cells were added to each well of 6 well plates. The plate was incubated in 37°C, 5% CO_2 incubator for 24 to 48 hours to achieve 90-100 % confluent growth in a single layer. The serum samples were heat-inactivated for 30 min at 56 °C and two-fold serially diluted using DMEM (Thermo Fisher Scientific) with a starting dilution of 1:10. WHO standard was used as positive control, and DMEM was used as negative control. The mixture was incubated in a 37° C water bath for 1 hour. 1 mL of each diluted sample/viral mixture was added in duplicate to each well, gently. The infected monolayer wells were incubated at 37°C, 5% CO_2 for 2 hours with occasional shaking of plates. The media of infected monolayer well were aspirated out. 2 mL of the agar/media mixture (final agarose concentration 0.6%) was gently added to each well for 6 well plates. The plate wells were incubated at 37°C,

5% CO_2 for 3-5 days. The cells were fixed with 1-2 mL of 4% formaldehyde at room temperature for 1 hour. The formaldehyde and agar/media were discarded, and the cells were washed with water. 0.25 to 0.5 mL of 0.5% crystal violet was added for 5 minutes. The number of plaques were counted using colony counter. Titers at which 50% reduction of plaques in compared to the negative control is taken as antibody titers. If the 50% reduction lies between the two dilution of serum sample, lowest difference is taken as antibody titers.

Microneutralization assay:

Neutralizing titers of serum samples were measured by the 50% tissue culture infectious dose ($TCID_{50}$) endpoint dilution assay with the BA.5 and XBB.1.9 variants[1]. Briefly, heat-inactivated sera were 2-fold serially diluted in DMEM medium with a starting dilution of 1:4 in 96-well plates in duplicate. The diluted serum was incubated with 100-150 $TCID_{50}$ of virus at 37 °C for 2 h, after which the serum virus mixtures were added with Vero E6 cell (Cell Bank, Chinese Academy of Sciences) suspension at a density of 2×10^4 cells per well. A control with non-infected cells, a negative serum and a positive serum control were included in each assay. The plates were incubated at 37 °C for about 96 h. Then cytopathic effects (CPE) of each well were recorded and $TCID_{50}$ values of each serum were determined.

Pseudovirus-based neutralization assay:

The SARS-CoV-2 pseudovirus based neutralization assay was performed as described previously[2,3]. In brief, the serum samples were first heat-inactivated at 56°C for 30 min. Serial 3-fold diluted serum, starting at 1:10, were incubated with 13,000 TCID50 of the pseudovirus (Vazyme Biotech Co., Ltd) for 1 hour at 37 °C, and then were inoculated with 2×10^4 Huh7 cells (100 μL) at 37 °C. After another 20 h of incubation, the luciferase (PerkinElmer) substrate was added to each well Luciferase activity was then measured using GloMax 96 Microplate Luminometer (Promega). The 50% neutralization titers (NT_{50}) were defined as the serum dilution at which the relative light units (RLUs) were reduced by 50% compared with the virus control wells. The NT_{50} was determined by non-linear regression, i.e., log (inhibitor) vs. normalized response (Variable slope), using GraphPad Prism 9.0 (GraphPad Software).

Statistical analyses

All data were analyzed with GraphPad Prism 9.0 software. Final results were illustrated via GMT with 95% CI (Geometric mean titers with 95% credibility interval). Statistical significance was determined using the unpaired t-test (Table S1 and S2) or multiple unpaired t-tests (Table S3).

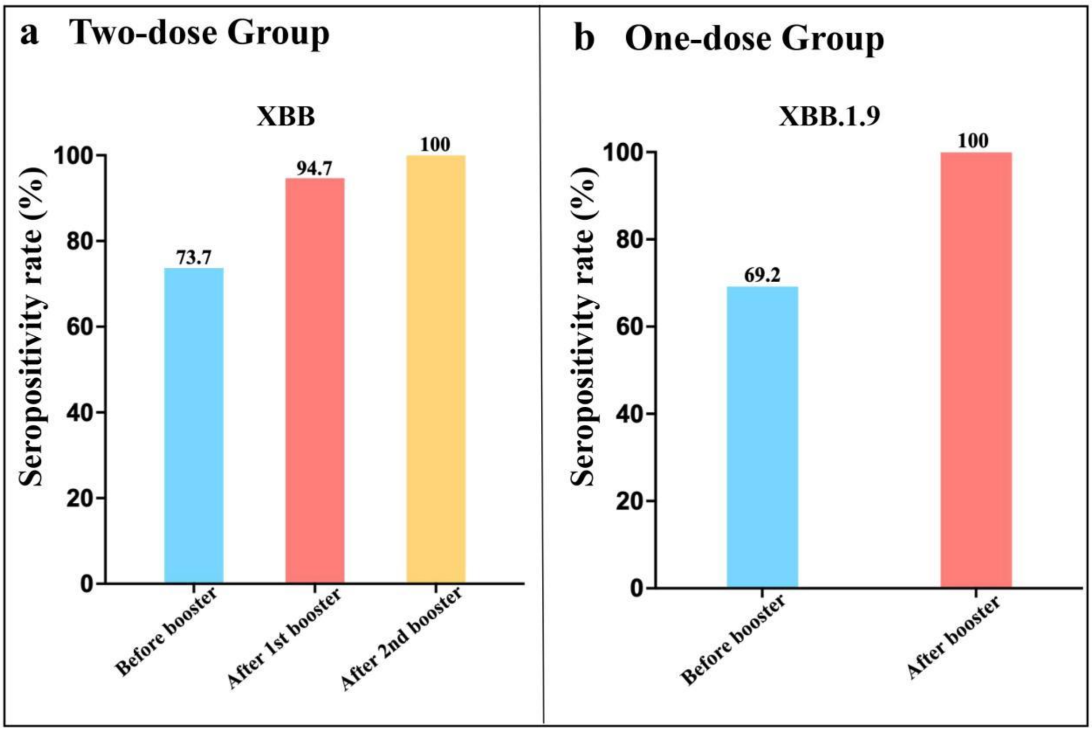

Supplementary Fig. S1 Seropositivity rate of neutralization antibody against XBB variants before and after the monovalent BA.4/5 mRNA vaccine (ABO1020) booster.

Panel a shows seropositivity rate of neutralization antibody against omicron subvariants XBB after two-dose booster of ABO1020. The seropositivity rate was assessed on day 0 before booster and day 28 after each booster (1st and 2nd booster), respectively. Panel b shows seropositivity rate of neutralization antibody against omicron subvariants XBB.1.9 after a single booster of ABO1020. The seropositivity rate was assessed on day 0 before booster and day 14 after booster, respectively.

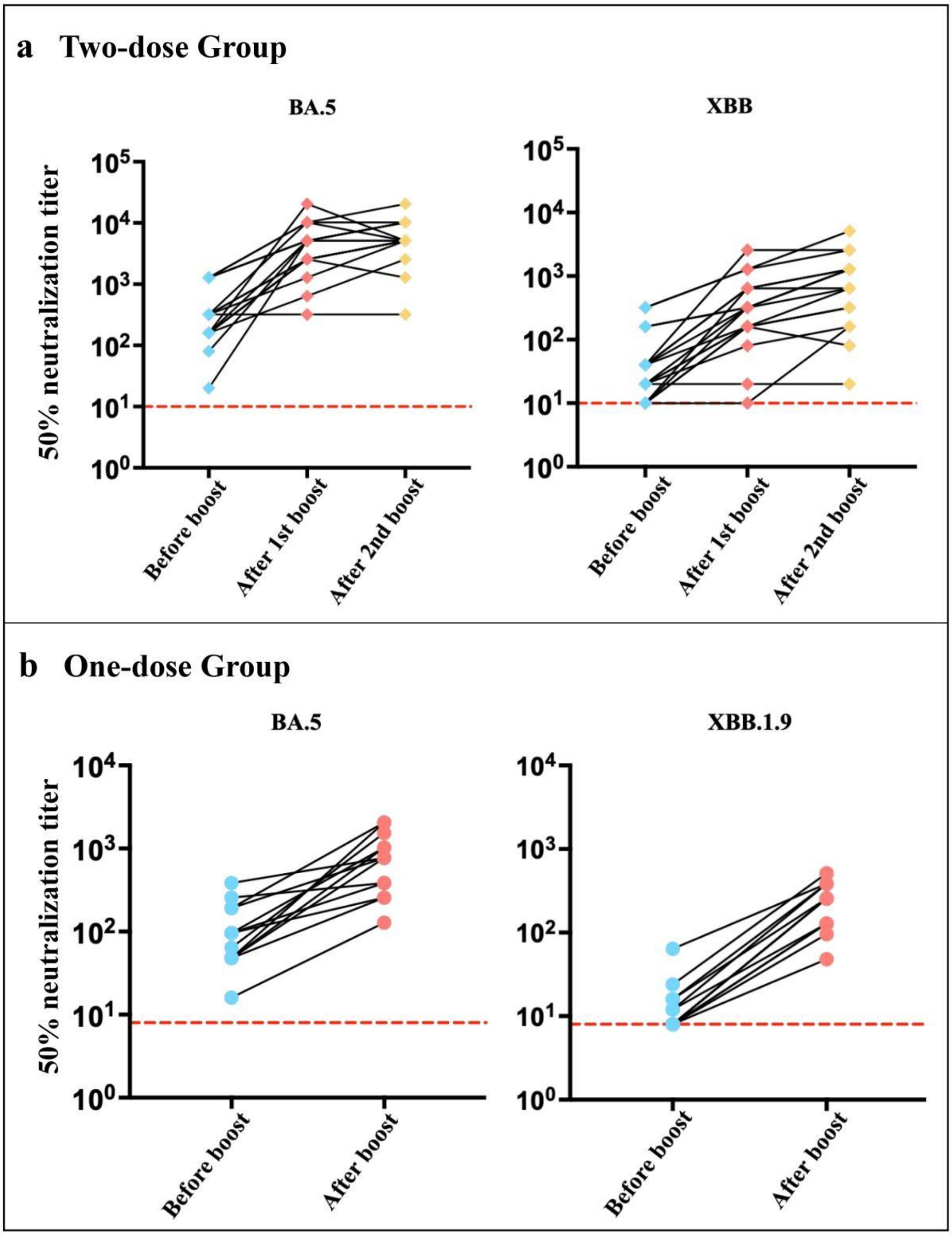

Supplementary Fig. S2 The neutralization titers of paired sera before and after the monovalent BA.4/5 mRNA vaccine (ABO1020) booster.

Panel a shows the 50% authentic virus neutralization titers against omicron subvariants BA.5 and XBB after two-dose booster of ABO1020. The neutralizing antibody titers were assessed on day 0 before booster and day 28 after each booster (1st and 2nd boost), respectively. Panel b shows the 50% authentic virus neutralization titers against omicron subvariants BA.5 and XBB.1.9 after a single booster of ABO1020. The neutralizing antibody titers were assessed on day 0 before booster and day 14 after booster, respectively. The dashed horizontal line represents the lower limit of detection.

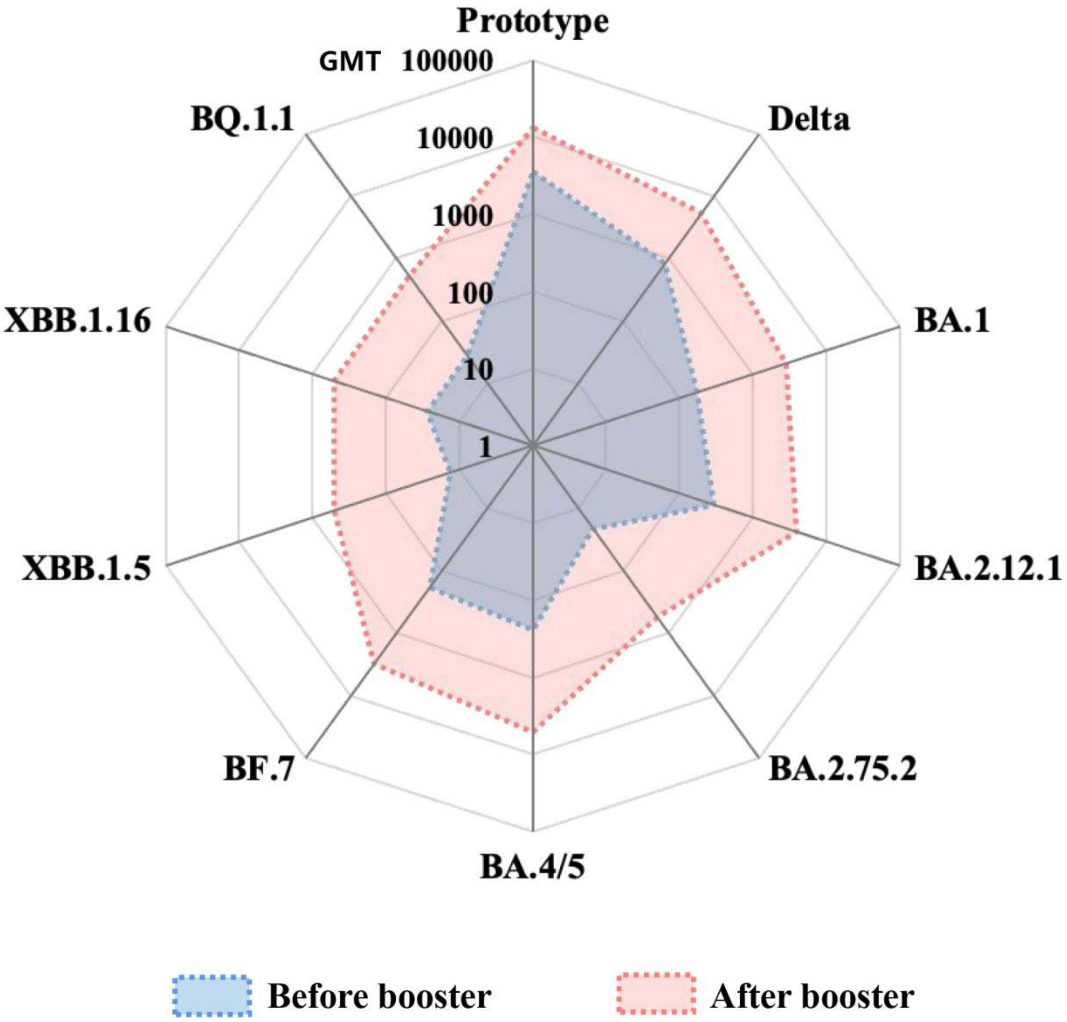

Supplementary Fig. S3 Radar plots of sera neutralization potency profile against prototype strain, Delta and Omicron subvariants before and after the monovalent BA.4/5 mRNA vaccine (ABO1020) booster in the pseudovirus analysis.

The radar plots show the 50% neutralization antibody titers of sera against different SARS-CoV-2 variants, including prototype strains, Delta and Omicron subvariants (BA.1, BA.2.12.1, BA.2.75.2, BA.4/5, BF.7, XBB.1.5, XBB.1.16, BQ.1.1) on day 0 before booster and day 14 after booster in a single booster of ABO1020. Average antibody levels are represented by geometric mean titers (GMT).

Supplementary Table S1 Demographics information and 50% neutralization titers of subjects received two doses of the monovalent BA.4/5 mRNA vaccine (ABO1020) booster.

ID	AGE (years)	SEX	Time since last vaccine (months)	*50% neutralization titers					
				BA.5			XBB		
				∮Before	§1st	§2nd	Before	1st	2nd
8010003	27	M	13.8	1280	10240	5120	320	1280	2560
8010007	24	M	9.6	320	1280	5120	10	160	80
8010008	36	M	18.2	160	2560	^N/A	20	160	N/A
8010009	31	M	17.0	160	2560	5120	20	160	320
8010011	20	M	15.0	160	5120	5120	20	80	160
8010013	27	M	22.3	1280	5120	5120	160	320	640
8010014	23	M	9.2	320	2560	1280	40	160	320
8010015	28	M	12.2	320	320	320	20	20	20
8010017	34	M	13.8	320	10240	10240	40	640	1280
8010022	26	M	11.6	80	5120	10240	10	320	1280
8010026	27	M	13.6	160	20480	5120	40	2560	2560
8010029	21	M	14.9	160	640	2560	10	10	160
8010034	23	M	17.8	320	2560	5120	10	160	640
8010037	24	M	15.0	1280	10240	20480	320	1280	5120
8010039	28	M	17.7	1280	5120	10240	160	320	1280
8010040	24	M	17.3	160	2560	N/A	20	320	N/A
8010043	28	M	11.7	160	2560	5120	40	640	640
8010044	30	M	13.1	20	5120	5120	10	640	1280
8010045	28	M	16.7	1280	5120	10240	40	320	1280

* Individual value of 50% neutralization titers is the geometric mean of duplicate results.

∮ Day 0 before booster (Before)

§ Day 28 after 1st or 2nd booster (1st, 2nd).

^ Not available (N/A).

Supplementary Table S2 Demographics information and 50% neutralization titers of subjects received a single monovalent BA.4/5 mRNA vaccine (ABO1020) booster.

ID	AGE (years)	SEX	Time since last vaccine (months)	*50% neutralization titers			
				BA.5		XBB.1.9	
				∮Before	§After	Before	After
8010051	44	F	15.3	256	384	12	384
8010052	31	F	16.9	192	2048	24	512
8010053	27	F	17.1	96	256	64	384
8010054	24	M	12.6	96	1024	<8	256
8010055	23	F	15.2	16	128	<8	96
8010056	47	F	17.8	192	768	12	128
8010058	33	M	17.9	96	384	8	256
8010059	22	F	18.1	384	768	16	384
8010060	25	M	15.1	48	1024	16	256
8010061	31	F	12.5	48	256	<8	48
8010062	33	M	24.4	48	2048	8	128
8010063	27	M	7.4	48	768	<8	128
8010064	34	F	7.7	64	1536	8	256

* Individual value of 50% neutralization titers is the geometric mean of duplicate results.

∮ Day 0 before booster (Before).

§ Day 14 after a single booster (After).

Supplementary Table S3 50% neutralization titers (pseudovirus) of subjects received a single monovalent BA.4/5 mRNA vaccine (ABO1020) booster.

ID	*50% neutralization titers (pseudovirus)																			
	Prototype		Delta		BA.1		BA.2.12.1		BA.2.75.2		BA.4/5		BF.7		XBB.1.5		XBB.1.16		BQ.1.1	
	∮Before	§After	Before	After	Before	After	Before	After	Before	After	Before	After	Before	After	Before	After	Before	After	Before	After
8010051	7695	17841	1343	10810	268.1	3385	2259	9140	34.58	579.6	3023	6489	2369	6356	10.46	907.4	36.77	790.7	193.1	3002
8010052	11893	70455	13651	20582	2313	10810	2198	13917	316.7	2832	2443	11689	1558	12387	31.01	1113	133.5	1555	60.51	1425
8010053	5474	13411	744.4	4256	456.1	1982	267	1279	69.97	305	208.6	3030	241.2	1196	71.57	577	236	820.3	58.97	201.1
8010054	3077	9494	902.3	6627	159.6	1956	688.2	2979	10.62	280.4	293	2946	265.5	3111	10	735	9.787	332.5	10.75	284.9
8010055	279.4	7491	15.1	3643	10	1824	10	905	10	90.16	10	1072	10	205.4	10	91.25	10	102.8	10	75.1
8010056	4935	14707	1739	6645	279.2	1416	579.3	5909	10.88	340.5	544.7	5575	448.9	2892	10	718.8	16.06	775	13.87	172.3
8010058	3372	5876	1010	2394	259.3	2929	129.5	2541	16.55	375.4	140.2	3371	120.,6	1468	10	318.2	19.83	462.6	14.95	567
8010059	11371	17572	6816	4743	1540	2143	1425	2422	182.6	1130	1780	10787	2279	6017	25.16	894	51.71	305.5	619.7	247.9
8010060	4990	15871	1275	2546	35.09	2938	74.72	5690	30.39	1890	89.25	8063	83.39	6833	10.26	1218	43.29	1758	52.05	1532
8010061	3789	10641	445.8	3839	302.8	1936	347.3	1071	19.95	310.2	311	2932	178.1	2855	10	79.69	15.39	136.7	73.65	351.6
8010062	3308	25810	1870	20704	854.9	9521	613.8	13842	10	3372	95.88	45129	54.47	14153	10	1152	28.58	921.5	19.72	1952
8010063	1947	8992	400.7	2669	41.22	1145	244.1	5041	10	529	167.5	4125	134.1	3075	10	377.6	10	140.8	10	440.3
8010064	4513	7473	900.2	2499	67.22	3793	169.3	5327	10.17	517.6	269.7	4389	155.7	2541	10	834.2	21.87	810	17.01	559.3

*Individual value of 50% neutralization titers (pseudovirus) is the geometric mean of duplicate results.

∮ Day 0 before booster (Before).

§ Day 14 after a single booster (After).

References

1. Wang Z, Zhao Z, Cui T, et al. Heterologous boosting with third dose of coronavirus disease recombinant subunit vaccine increases neutralizing antibodies and T cell immunity against different severe acute respiratory syndrome coronavirus 2 variants. Emerg Microbes Infect 2022;11:829-40.
2. Nie J, Li Q, Wu J, et al. Quantification of SARS-CoV-2 neutralizing antibody by a pseudotyped virus-based assay. Nat Protoc 2020;15:3699-715.
3. Zhang NN, Li XF, Deng YQ, et al. A Thermostable mRNA Vaccine against COVID-19. Cell 2020;182:1271-83 e16.
